# Rapamycin extends lifespan and delays tumorigenesis in heterozygous p53+/− mice

**DOI:** 10.18632/aging.100498

**Published:** 2012-10-29

**Authors:** Elena A. Komarova, Marina P. Antoch, Liliya R. Novototskaya, Olga B. Chernova, Geraldine Paszkiewicz, Olga V. Leontieva, Mikhail V. Blagosklonny, Andrei V. Gudkov

**Affiliations:** ^1^ Department of Cell Stress Biology, Roswell Park Cancer Institute, BLSC, L3-312, Buffalo, NY 14263, USA; ^2^ Department of Molecular & Cellular Biology, Roswell Park Cancer Institute, BLSC, L3-312, Buffalo, NY 14263, USA; ^3^ Tartis Aging, Inc., Buffalo, NY 14203, USA

**Keywords:** cancer, mutations, DNA damage, aging, mTOR

## Abstract

The TOR (Target of Rapamycin) pathway accelerates cellular and organismal aging. Similar to rapamycin, p53 can inhibit the mTOR pathway in some mammalian cells. Mice lacking one copy of p53 (p53+/− mice) have an increased cancer incidence and a shorter lifespan. We hypothesize that rapamycin can delay cancer in heterozygous p53+/− mice. Here we show that rapamycin (given in a drinking water) extended the mean lifespan of p53+/− mice by 10% and when treatment started early in life (at the age less than 5 months) by 28%. In addition, rapamycin decreased the incidence of spontaneous tumors. This observation may have applications in management of Li-Fraumeni syndrome patients characterized by heterozygous mutations in the *p53* gene.

## INTRODUCTION

The mTOR (mammalian Target of Rapamycin) pathway plays a crucial role in the geroconversion from cell cycle arrest to senescence (geroconversion) [1]. Rapamycin suppresses or decelerates geroconversion, maintaining quiescence instead [2-8]. Furthemore, inhibition of the TOR pathway prolongs lifespan in model organisms, including mice [9-13]. In an organism, nutrients activate mTOR [14-16], whereas fasting or calorie restriction deactivates mTOR [17-19]. Calorie restriction slows down aging [20] and postpones tumorigenesis in several animal models [21,22], including p53-deficient mice [23-25].

Similar to other tumor suppressors, p53 can inhibit mTOR in mammalian cells [26-31]. While causing cell cycle arrest, p53 can suppress geroconversion, thus preventing a senescent phenotype in the arrested cells [30,31]. Therefore, it is not suprising that p53 inhibits hyper-secretory phenotype, a hallmark of senescence [32] whereas p53-deficiency resulted in pro-inflammatory phenotype [33,34]. Noteworthy, the activity of p53 is decreased with aging [35]. Lack of one p53-allele (p53+/−) accelerates carcinogenesis and shortens lifespan [36-41]. We propose that rapamycin can decelerate cancer development in p53+/− mice. Here we show experimental evidence supporting this hypothesis.

## RESULTS

**Figure 1:**
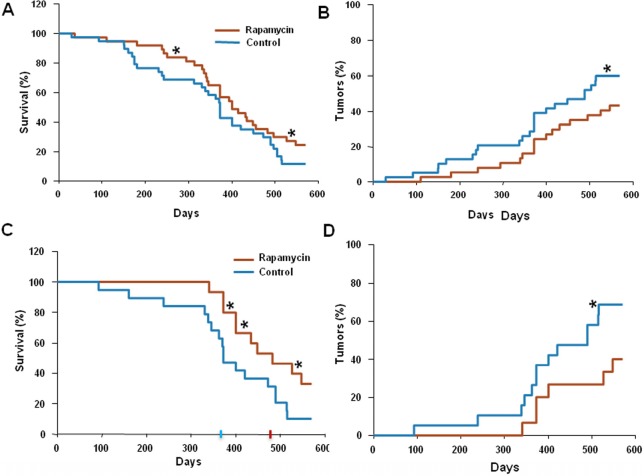
Administration of rapamycin extends lifespan and delays carcinogenesis in p53+/− male mice (**A**) Kaplan Meier survival curve of rapamycin-treated (red line) and control (blue line) mice. (**B**) Incidence of tumors in rapamycin-treated (red) and control (blue) mice. Animals received rapamycin starting at various ages at 1.5 mg/kg per day in drinking water throughout entire life. * p<0.05. (**C**) Kaplan Meier survival curve of rapamycin-treated (red line) and control (blue line) mice that start receiving rapamycin early in life (<5 months). (**D**) Incidence of tumors in rapamycin-treated (red) and control (blue) mice that start receiving rapamycin early in life (<5 months). * p<0.05 toph

Rapamycin (approximate dose, 1.5 mg/kg/day) was given in drinking water. 75 mice were divided into two groups: control (n=38) and rapamycin-treated (n=37). The mean lifespan of animals in control group was 373 days and the last 10% of survivals lived as long as 520 days (Fig. 1 A). In rapamycin-treated mice, the mean lifespan was 410 days and lifespan of the last 10% of survivals could not be determined (Fig. 1 A). Mice in both groups were also monitored for tumor development. The data presented in Fig. 1B demonstrate that carcinogenesis was significantly delayed in rapamycin-treated mice compared to control mice.

Since in our experiments animals started to receive rapamycin at different age, we sought to test whether this affected the outcome of the treatment.

For this, we further subdivided all mice used into two groups: “young” (receiving rapamycin from the age of 5 months or earlier) and “old” (receiving rapamycin starting at 5 months of age or older). Results of the data analysis for the “young” group are shown in Figure 1C and D. The mean lifespan in control group was 373 days, whereas in rapamycin-treated “young” mice the mean lifespan reached 480 days, 3.5 months increase over the control group. Furthermore, 40% of rapamycin-treated “young” mice survived 550 days (Fig. 1C) and by this age developed 2 times less tumors than control mice (Fig. 1D). In the “old” group the difference between control and treated group was blunted (data not shown).

**Figure 2:**
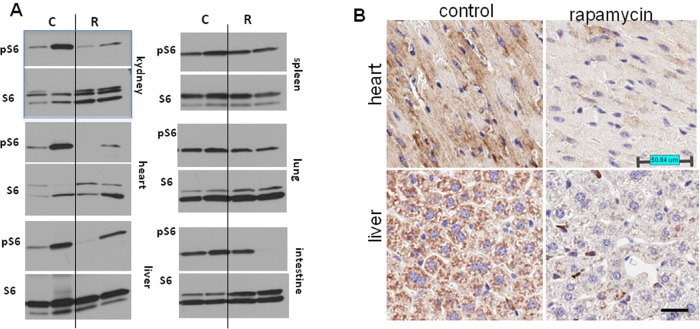
Administration of rapamycin in drinking water inhibits the mTOR pathway in p53+/− male mice (**A**) Western blot analysis of whole cell lysates of 6 organs of rapamycin-treated and control mice probed with antibodies specific to S6 and phospho-S6 (Ser240/244). Mice received rapamycin in drinking water for 2 days. (**B**) Immunohistochemistry. pS6 in the heart and the liver. Mice received rapamycin in drinking water for 2 days.

Thus, the life-extending effect of rapamycin is more pronounced when treatment starts earlier in life. In order to confirm that rapamycin administered with drinking water has biological activity in vivo, we measured levels of phosphorylated ribosomal protein S6 (pS6), a marker of the mTOR activity in tissues of control and rapamycin-treated mice. After receiving rapamycin in drinking water for 2 days, mice were sacrificed and the levels of total S6 and pS6 were estimated by Western blot analysis and immuno-cytochemistry (Fig. 2).

As shown in Fig. 2A, levels of pS6 were reduced in the heart, kidney and liver of rapamycin-treated mice. Also, pS6/S6 ratios were lower in rapamycin-treated mice (Fig. S1).

These results were confirmed by immunohistochemical staining showing lower levels of pS6 in tissues of rapamycin-treated mice (Fig. 2B). The variability of pS6 levels among mice may explain the variability of biological effects of rapamycin.

## DISCUSSION

Previously it was shown that rapamycin prolongs lifespan in genetically heterogeneous mice [11], [12], inbred mice [42] and Her2-expressing mice [13]. In normal genetically heterogeneous mice, rapamycin extended life span even when its administration was started later in life [11]. Our data in p53+/− mice show that the effect of rapamycin was blunted when treatment started at the age of 5 months or older.

This indicates that the anti-cancer effect of rapamycin is likely to be indirect and is imposed via its systemic effect at the level of an organism rather than through direct inhibition of tumor growth. To further address this question we plan to test the effect of rapamycin on animals with established tumors (by measuring tumor growth) along with evaluating the functional status of mTOR and the ability of rapamycin to suppress it in tumors and normal tissues. As we report here, administration of rapamycin starting early in life increased mean lifespan in p53+/− male mice by 28%. Previous work has demonstrated that the life-extending effects of rapamycin [11,12] as well as metformin [43,44], calorie restriction [45] and genetic inhibition of the IGF-I/mTOR/S6K pathway [46,47] were less pronounced in male mice compared with female mice. Moreover, in some cases, life span extension was achieved in female mice only [43,47]. Therefore, the observed increase in the median lifespan is dramatic, taking into account that it was achieved in male mice. However, because of low bioavailability of rapamycin, it was given constantly (in drinking water) without interruptions, whereas intermittent schedules may be more appropriate for future clinical developments as cancer-preventive interventions. In fact, a novel formulation of rapamycin (Rapatar) may be given intermittently, which still reveal even more pronounced extension of life span in p53-deficient mice (Comas et al, Aging 2012; this issue).

Our study suggests that rapamycin can be considered for cancer prevention in patients with Li-Fraumeni syndrome. Li-Fraumeni syndrome is an autosomal dominant disorder with a germline p53 mutation [48]. The incidence of cancer in carriers of mutation reaches 50% at the age of 40 and 90% at the age 60. Children of affected parents have an approximate 50% risk of inheriting the familial mutation [48]. Although functional assays have been established allowing for easy genetic testing for TP53 mutation, no effective chemopreventive therapy is currently available. The p53 rescue compounds may hold some promise in the future [48-50]; however these are not clinically approved drugs. In contrast, rapamycin has been used in the clinic for over a decade mostly in renal transplant patients. It was reported that rapamycin significantly decreased cancer incidence in renal transplant patients [51-53]. Our data suggest that rapamycin or its analogs can be considered for cancer prevention in Li-Fraumeni syndrome.

## METHODS

### Mice

All animal studies were conducted in accordance with the regulations of the Committee of Animal Care and Use at Roswell Park Cancer Institute. The colony of p53-knockout mice on a C57B1/6 background (originally obtained from Jackson Laboratories, Bar Habor, ME) was maintained by crossing p53+/− females with p53−/− males followed by genotyping of the progeny (PCR) as described previously [54]. Heterozygous p53+/− mice were generated by crossing p53−/− males with wild type p53 females. Male mice were kept in polypropelene cages (30×21×10 cm) under standard light/dark regimen (12 hours light: 12 hours darkness) at 22 ± 2°C, And received standard laboratory chow and water ad libitum.

### Rapamycin treatment

Rapamycin (LC Laboratories, USA) was diluted in ethanol at concentration 15 mg/ml. Then the stock was diluted 1:1000 in drinking water. Drinking water was changed every week. Male mice were randomly divided into two groups. Mice of the first group (n=37) were given rapamycin in drinking water (approximately 1.5 mg/kg per day), whereas mice of the second group (n=38) were given tap water without rapamycin and served as control. Once a week all mice were palpated for detection of tumor mass appearance.

### Pathomorphological examination

All animals were autopsied. Site, number and size of tumors were checked. All tumors, as well as the tissues and organs with suspected tumor development were excised and fixed in 10% neutral formalin. After the routine histological processing the tissues were embedded into paraffin. 5–7 μm thin histological sections were stained with haematoxylin and eosine and were microscopically examined. Tumors were classified according to International Agency for Research on Cancer recommendations.

### Western blot analysis

Tissues were homogenized in Bullet blender using stainless steel 0.5 mm diameter beads (Next Advantage, Inc. NY, USA) and RIPA lysis buffer supplemented with protease and phosphatase inhibitors tablets (Roche Diagnostics, Indianopolis, IN, USA). Lysates were cleared by centrifugation at 4°C at 13000 rpm. Equal amounts of protein were separated on gradient Criterion gels (BioRad) and immunoblotting was performed with rabbit anti-phospho S6 (Ser 240/244) and mouse anti-S6 antibodies from Cell Signaling Biotechnology as described previously [55], [56].

### Immunochemistry

Dissected tissue samples were fixed in 10% buffered formalin, embedded into paraffin. 5–7 μm thin histological sections were stained with anti-phospho S6 (Ser240/244) antibody (Cell Signaling) and counterstained with Hematoxylin.

### Statistical analyses

The SigmaStat software package was used for analysis. The P values were calculated using Fisher's Exact Test (2-tail). P<0.05 was considered as statistically significant.

## SUPPLEMENTAL FIGURE


