# Supplementary material for: Rapamycin extends lifespan and delays tumorigenesis in heterozygous p53+/− mice
Source: Aging (Albany NY). 2012 Oct 29;4(10):709–14. doi: 10.18632/aging.100498 (PMC3517941; doi:10.18632/aging.100498)
Supplement: Supplementary file 1 [file aging-04-709-s001.pdf]

Blagosklonny MV. Rapamycin increases lifespan and inhibits spontaneous tumorigenesis in inbred female mice. Cell Cycle. 2011; 10: 4230-4236.
43. Anisimov VN, Piskunova TS, Popovich IG, Zabezhinski MA, Tyndyk ML, Egormin PA, Yurova MV, Rosenfeld SV, Semenchenko AV, Kovalenko IG, Poroshina TE, Berstein LM. Gender differences in metformin effect on aging, life span and spontaneous tumorigenesis in 129/Sv mice. Aging (Albany NY). 2010; 2: 945-958.
44. Blagosklonny MV. Metformin and sex: Why suppression of aging may be harmful to young male mice. Aging (Albany NY). 2010; 2: 897-899.
45. Turturro A, Duffy P, Hass B, Kodell R, Hart R. Survival characteristics and age-adjusted disease incidences in C57BL/6 mice fed a commonly used cereal-based diet modulated by dietary restriction. J Gerontol A Biol Sci Med Sci. 2002; 57: B379-389.
46. Holzenberger M, Dupont J, Ducos B, Leneuve P, Geloen A, Even PC, Cervera P, Le Bouc Y. IGF-1 receptor regulates lifespan and resistance to oxidative stress in mice. Nature. 2003; 421: 182-187.
47. Selman C, Tullet JM, Wieser D, Irvine E, Lingard SJ, Choudhury AI, Claret M, Al-Qassab H, Carmignac D, Ramadani F, Woods A, Robinson IC, Schuster E, Batterham RL, Kozma SC, Thomas G et al. Ribosomal protein S6 kinase 1 signaling regulates mammalian life span. Science. 2009; 326: 140-144.
48. Upton B, Chu Q, Li BD. Li-Fraumeni syndrome: the genetics and treatment considerations for the sarcoma and associated neoplasms. Surg Oncol Clin N Am. 2009; 18: 145-156, ix.
49. Glazer RI. A new therapeutic basis for treating Li-Fraumeni Syndrome breast tumors expressing mutated TP53. Oncotarget. 2010; 1: 470-471.
50. Herbert BS, Chanoux RA, Liu Y, Baenziger PH, Goswami CP, McClintick JN, Edenberg HJ, Pennington RE, Lipkin SM, Kopelovich L. A molecular signature of normal breast epithelial and stromal cells from Li-Fraumeni syndrome mutation carriers. Oncotarget. 2010; 1: 405-422.
51. Mathew T, Kreis H, Friend P. Two-year incidence of malignancy in sirolimus-treated renal transplant recipients: results from five multicenter studies. Clin Transplant. 2004; 18: 446-449.
52. Campistol JM, Eris J, Oberbauer R, Friend P, Hutchison B, Morales JM, Claesson K, Stallone G, Russ G, Rostaing L, Kreis H, Burke JT, Brault Y, Scarola JA, Neylan JF. Sirolimus Therapy after Early Cyclosporine Withdrawal Reduces the Risk for Cancer in Adult Renal Transplantation. J Am Soc Nephrol. 2006; 17: 581-589.
53. Blagosklonny MV. Prevention of cancer by inhibiting aging. Cancer Biol Ther. 2008; 7: 1520-1524.
54. Leonova KI, Shneyder J, Antoch MP, Toshkov IA, Novototskaya LR, Komarov PG, Komarova EA, Gudkov AV. A small molecule inhibitor of p53 stimulates amplification of hematopoietic stem cells but does not promote tumor development in mice. Cell Cycle. 2010; 9: 1434-1443.
55. Leontieva O, Gudkov A, Blagosklonny M. Weak p53 permits senescence during cell cycle arrest. Cell Cycle. 2010; 9: 4323-4327.
56. Leontieva OV, Blagosklonny MV. DNA damaging agents and p53 do not cause senescence in quiescent cells, while consecutive re-activation of mTOR is associated with conversion to senescence. Aging (Albany NY). 2010; 2: 924-935.

SUPPLEMENTAL FIGURE

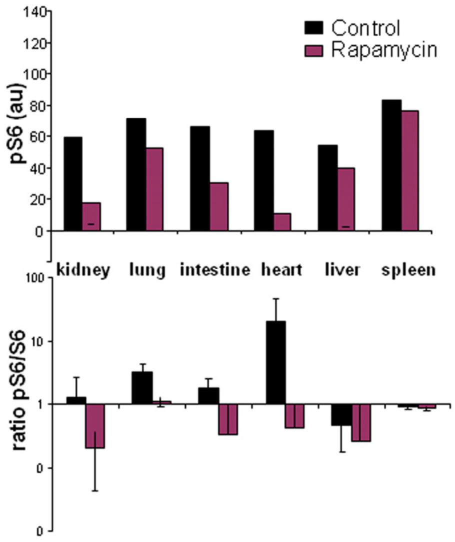

Supplemental Figure S1. Quantitative analysis of data shown in Figure 2A. Top panel - Intensity of phosphorylated S6 (pS6) signal was quantified using ImageJ program (intensity units, IU). Bottom panel – Intensity of pS6 and S6 signals were quantified and the ratio pS6/S6 was calculated.
